# The lifelong orgasm gap: exploring age’s impact on orgasm rates

**DOI:** 10.1093/sexmed/qfae042

**Published:** 2024-07-01

**Authors:** Amanda N Gesselman, Margaret Bennett-Brown, Simon Dubé, Ellen M Kaufman, Jessica T Campbell, Justin R Garcia

**Affiliations:** The Kinsey Institute, Indiana University, Bloomington, IN 47405, United States; The Kinsey Institute, Indiana University, Bloomington, IN 47405, United States; Department of Communication Studies, Texas Tech University, Lubbock, TX 79409, United States; The Kinsey Institute, Indiana University, Bloomington, IN 47405, United States; Department of Psychology, Concordia University, Montreal, Quebec, H4B 1R6, Canada; The Kinsey Institute, Indiana University, Bloomington, IN 47405, United States; The Kinsey Institute, Indiana University, Bloomington, IN 47405, United States; The Kinsey Institute, Indiana University, Bloomington, IN 47405, United States; Department of Gender Studies, Indiana University, Bloomington, IN 47405, United States

**Keywords:** orgasm, aging, orgasm gap, sexual orientation, gender differences

## Abstract

**Background:**

Research demonstrates significant gender- and sexual orientation–based differences in orgasm rates from sexual intercourse; however, this “orgasm gap” has not been studied with respect to age.

**Aim:**

The study sought to examine age-related disparities in orgasm rates from sexual intercourse by gender and sexual orientation.

**Methods:**

A survey sample of 24 752 adults from the United States, ranging in age from 18 to 100 years. Data were collected across 8 cross-sectional surveys between 2015 and 2023.

**Outcomes:**

Participants reported their average rate of orgasm during sexual intercourse, from 0% to 100%.

**Results:**

Orgasm rate was associated with age but with minimal effect size. In all age groups, men reported higher rates of orgasm than did women. Men’s orgasm rates ranged from 70% to 85%, while women’s ranged from 46% to 58%. Men reported orgasm rates between 22% and 30% higher than women’s rates. Sexual orientation impacted orgasm rates by gender but not uniformly across age groups.

**Clinical Translation:**

The persistence of the orgasm gap across ages necessitates a tailored approach in clinical practice and education, focusing on inclusive sexual health discussions, addressing the unique challenges of sexual minorities and aging, and emphasizing mutual satisfaction to promote sexual well-being for all.

**Strengths and Limitations:**

This study is the first to examine the orgasm gap with respect to age, and does so in a large, diverse sample. Findings are limited by methodology, including single-item assessments of orgasm and a sample of single adults.

**Conclusion:**

This study revealed enduring disparities in orgasm rates from sexual intercourse, likely resulting from many factors, including sociocultural norms and inadequate sex education.

Orgasm is a psychophysiological response to sexual stimulation involving muscle contractions, hormonal changes, and tension release.^1^ While not essential for sexual pleasure,^2^^,^^3^ orgasms are frequently sought in both solitary and partnered sexual activity, contributing to sexual satisfaction and well-being.^4-8^ The absence of orgasm can cause distress and relationship challenges.^9-11^ In sexual intercourse, orgasm rates vary across genders and sexual orientations, with women—particularly heterosexual women—reporting lower orgasm rates than men and lesbian women (ie, the orgasm gap).^12-14^ Prior investigations into these disparities have revealed a complex interplay of biological, psychological, and sociocultural factors influencing sexual pleasure. However, the impact of age on the orgasm gap is unexplored. Studying age in this context can uncover developmental influences on sexual health, offering insights for interventions that enhance sexual functioning across the lifespan. This study assessed differences in orgasm rates from sexual intercourse by gender, sexual orientation, and age.

## The orgasm gap

The orgasm gap between genders is substantial, with reported differences ranging from 25% to 52%.^12-20^ For example, a recent study found that 82% of men reported orgasming during their most recent casual sexual encounter compared with only 32% of women in the study.^20^ Factors contributing to this gap include physiological, anatomical, and sociocultural elements. For women, orgasm variations are partly due to anatomical differences, such as clitoral-vaginal distance, and responses to different types of stimulation.^21-24^ Hormonal fluctuations, like changes in estrogen and testosterone during the menstrual cycle and menopause, also affect women's libido and orgasmic capacity.^25-28^ Conversely, men’s sexual function is more consistently linked with testosterone levels, which can decline with age.^29-31^

Orgasm is influenced by interpersonal and sociocultural factors. Relationship dynamics and partner behaviors impact orgasm frequency, particularly for women. Open sexual communication and prioritization of foreplay by partners are positively associated with women’s orgasm.^32-34^ Sociocultural influences, including patriarchy, sexism, inadequate sexual education, and the cultural overvaluation of penetrative sex, contribute to orgasm discrepancies between young heterosexual men and women.^35^ These factors lead to disparities in pleasure-centric sexual behaviors, reinforcing the imbalance in orgasm rates.^4^^,^^36-38^ This bias might extend to sex education, in which male pleasure is emphasized more than female pleasure in heteronormative contexts.^39^

The association between gender and orgasm rates is often moderated by sexual orientation. While men’s sexual orientation does not seem to affect orgasm rates, lesbian women report higher orgasm rates than heterosexual women and perceive their partners as experiencing more orgasms than heterosexual men.^12-14^ Lesbian women are more likely to engage in and receive oral sex, with encounters often lasting longer than those of heterosexual women.^40^ These differences suggest an egalitarian approach to sexual pleasure among lesbian couples, contrasting with heterosexual dynamics. The disparity in orgasm rates, potentially rooted in differing expectations and expressions of sexual intimacy, emphasizes the need to understand how sexual orientation influences orgasm rates from intercourse.

Beyond gender and sexual orientation, age—and the intersection of age, gender, and sexual orientation—may also influence the orgasm gap. Aging introduces physiological and psychological shifts that can affect sexual behavior.^41-51^ For women, these challenges include hormonal changes impacting sexual functioning and desire. Older women are less likely than older men to engage in sexual activities or to report the desire to do so.^44^^,^^52^ Women also experience a decline in sexual thoughts earlier than men.^44^ Given that women’s orgasm rates are already lower than men’s, age may further widen the orgasm gap. However, older women report higher levels of sexual satisfaction compared with younger women,^52^ suggesting that sex may become more pleasurable with age, potentially leading to higher orgasm rates. This implies the orgasm gap between men and women may be smaller in younger age groups.

## Current study

We conducted a secondary analysis of data from the annual, cross-sectional Singles in America (SIA) study to examine orgasm rates from sexual intercourse and determine if the orgasm gap persists across adult age groups. Our sample includes nearly 25 000 single (ie, romantically unpartnered) participants, 18 to 100 years of age. While focusing on singles omits adults in romantic relationships, singles constitute approximately one-third of the U.S. adult population,^53^ making our findings broadly relevant. Sexual satisfaction, including orgasm, remains significant for singles, challenging the notion that orgasm is only relevant within romantic relationships.^54-56^ Despite focusing solely on singles, the sample’s size and diversity provide a unique perspective on the orgasm gap. This research is the first to explore age’s influence on the orgasm gap, offering insights for further exploration in diverse populations.

Given the lack of studies on the orgasm gap related to age, our study was exploratory, guided by 3 research questions: (1) Is age associated with orgasm rate?; (2) Is age significantly associated with gender and sexual orientation in predicting orgasm rate?; and (3) Does the orgasm gap exist in all adult age groups? These findings enhance our understanding of orgasm experiences throughout the lifespan and provide deeper insights into the factors influencing orgasm rates during sexual intercourse.

## Method

### Data collection

Data were collected in 2015 to 2017 and 2019 to 2023 as part of the SIA study; data were not collected in 2018 due to restricted resources. SIA is an annual, cross-sectional, online survey about single American adults’ romantic and sexual behaviors and perceptions. The online dating company Match develops the survey with input from academic researchers. While Match funds the study, participants are not recruited via match.com and inclusion criteria do not involve online dating. Participants are required to be legal adults (18+ years of age) and to be single, defined for participants as being both unmarried and not in a committed romantic relationship.

Participants were recruited by Dynata or ResearchNow using Internet research panels created for online surveying. Panelists are drawn from a pool of established participants recruited over several years from various venues (eg, paper and electronic mailings, corporate partnerships). Eligible panel members received a recruitment message from Dynata or ResearchNow that briefly described SIA and invited participation for financial compensation. All survey data were de-identified before being provided to the research team; thus, this research was exempt from further review by the Institutional Review Board according to federal regulations at 45 CFRP part 46, paragraph d, number four, item ii.^57^

### Data cleaning

**Figure 1:**
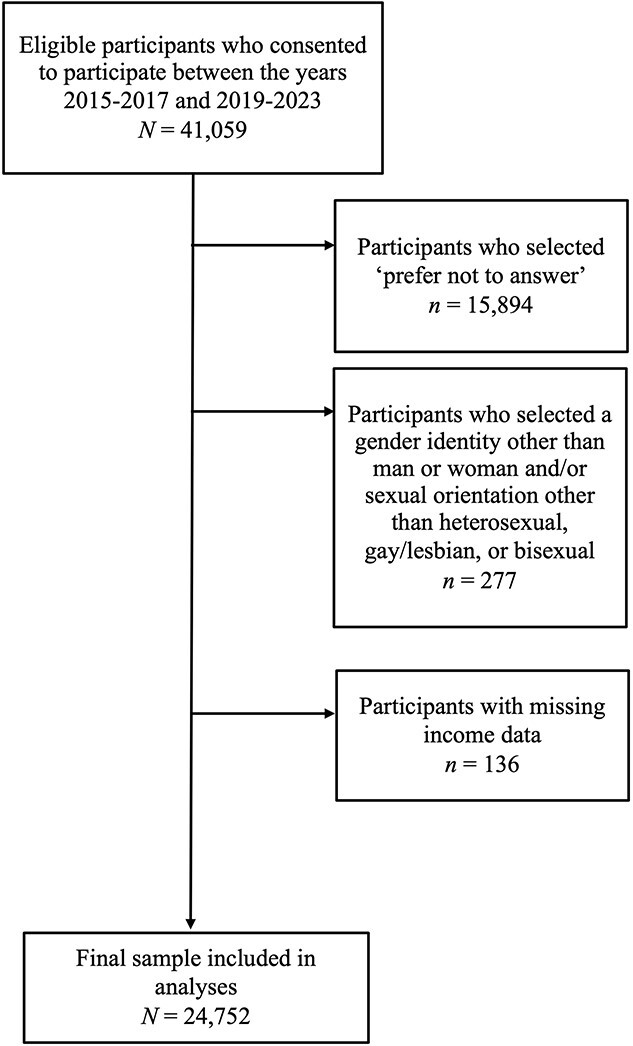
Participant exclusion criteria and final sample explanation.

The data were analyzed using IBM SPSS v29. The initial dataset included 41 059 participants. See Figure 1 for data cleaning details. Because prior research has shown an association between annual income and orgasm rates,^58^ we controlled for income in all analytic models; participants who did not report their income were removed prior to analyses. The final analytic sample consisted of 24 752 participants.

### Participants

The final analytic sample (N = 24 752) included between 2414 (in 2021) and 3500 (in 2017) participants from each survey year. The sample’s gender composition was 53.0% (n = 13 127) women and 47.0% (n = 11 625) men. Age ranged from 18 to 100 years (mean 44.50 ± 16.57 years). See Table 1 for participant demographics.

**Table 1:** Participant demographics for the final analytic sample (N = 24 752).

Gender	
Women	53.0 (13 127)
Men	47.0 (11 625)
Race/ethnic group	
White	61.7 (15 243)
Black/African-American	18.5 (4560)
Hispanic or Latino	11.8 (2917)
Asian	4.5 (1113)
Native American/Alaskan Native	1.8 (442)
Another race/ethnicity not listed	1.7 (430)
Sexual orientation	
Straight/heterosexual	86.6 (21 444)
Gay/lesbian	7.9 (1954)
Bisexual	5.5 (1354)
Annual household income	
<$15 000	15.1 (3733)
$15 000-$29 999	21.2 (5255)
$30 000-$44 999	18.7 (4634)
$45 000-$59 999	15.1 (3744)
$60 000-$74 999	10.1 (2496)
$75 000-$99 999	9.8 (2438)
$100 000-$149 999	6.8 (1692)
$150 000 or more	3.1 (760)
Age, y	44.50 ± 16.57

Values are % (n) or mean ± SD.

### Measures

#### Orgasm rate

Participants responded to the following question: “When having sexual intercourse in general, what percentage of the time do you usually have an orgasm?” Responses were made on a scale of 0% to 100%.

#### Demographics

We report demographics that were included in all years of SIA. All demographic and data collection information for the SIA surveys included here can be accessed at https://osf.io/xyg6e/. Participants reported age, income, gender, sexual orientation, and race/ethnicity.

### Data analysis

Because the data are cross-sectional, we collapsed data across all survey years, controlling for the effect of year in all analytic models. For research question 1 (RQ1) and RQ2, we conducted a linear regression with orgasm rate as the outcome variable. Predictors included the linear and curvilinear effects of age (mean centered), gender (women vs men), sexual orientation (2 contrast codes; code 1: heterosexual vs gay/lesbian/bisexual, code 2: gay/lesbian vs bisexual), and their 2- and 3-way interaction terms. Income and survey year (mean centered; both linear and curvilinear effects) were controlled variables.

For RQ 3, we categorized participants into 1 of 5 adult age groups derived from Erikson’s theorized stages of psychosocial development, as well as Arnett’s scholarship on emerging adulthood.^59^^,^^60^ Age groups were (1) emerging adults, 18 to 24 years of age; (2) young adults, 25 to 34 years of age; (3) early middle adults, 35 to 49 years of age; (4) late middle adults, 50 to 64 years of age; and (5) elder adults, 65 years of age and older. Within each group, we conducted a linear regression with orgasm rate as the outcome variable; gender, both sexual orientation variables, and their 2-way interactions were included as predictors, along with income and year (both linear and curvilinear effects) as controlled variables.

## Results

The average orgasm rate for the overall sample was 64.74 ± 36.26%.

### RQ1: Is age associated with orgasm rate?

Linear and curvilinear age variables significantly predicted orgasm rate, indicating both a positive linear association and a nonlinear association with age. However, very small effect sizes, combined with our large sample size, suggested that these associations were negligible (*r*_p_ = 0.04 and *r*_p_ = −0.01, respectively). See Table 2 for regression coefficients.

**Table 2:** Regression coefficients for the overall sample, including all age groups.

Variable	*b* (95% CI)	*t*	*r*_p_
Income	0.87 (0.66 to 1.08)	8.15^c^	0.05
Year, linear	−0.92 (−0.74 to −0.44)	−10.07^c^	−0.06
Year, nonlinear	0.37 (0.28 to 0.45)	8.18^c^	0.05
Gender	−24.10 (−26.11 to −22.09)	−23.50^c^	−0.13
Sexual orientation: heterosexual vs LGB	−7.20 (−10.19 to −4.21)	−4.73^c^	−0.03
Sexual orientation: gay/lesbian vs bisexual	4.41 (−1.21 to 10.02)	1.54	0.01
Age, linear	0.51 (0.37 to 0.65)	7.06^c^	0.04
Age, nonlinear	−0.01 (−0.02 to 0.001)	−2.00^a^	−0.01
Age × gender	−0.06 (−0.16 to 0.04)	−1.25	−0.01
Age × heterosexual vs LGB	−0.13 (−0.27 to 0.01)	−1.83	−0.01
Age × gay/lesbian vs bisexual	0.34 (0.07 to 0.61)	2.50^a^	0.01
Age^2^ × gender	−0.001 (−0.01 to 0.01)	−0.21	−0.00
Age^2^ × heterosexual vs LGB	0.01 (0.001 to 0.02)	2.05	0.01
Age^2^ × gay/lesbian vs bisexual	0.01 (−0.01 to 0.02)	1.24	0.01
Gender × heterosexual vs LGB	4.92 (3.03 to 7.04)	4.92^c^	0.03
Gender × gay/lesbian vs bisexual	−4.27 (−8.08 to −0.45)	−2.19^a^	−0.01
Age × gender × heterosexual vs LGB	0.07 (−0.03 to 0.17)	1.33	0.01
Age × gender × gay/lesbian vs bisexual	−0.26 (−0.45 to −0.07)	−2.70^b^	−0.02
Age^2^ × gender × heterosexual vs LGB	−0.004 (−0.01 to 0.001)	−0.06	−0.01
Age^2^ × gender × gay/lesbian vs bisexual	−0.01 (−0.02 to 0.004)	−1.15	−0.01

*R* ^2^ = 0.20, *R*^2^ adjusted = 0.20, *F*_20,24 731_ = 300.74, *p* < .001.

Abbreviations: CI, confidence interval; LGB, lesbian/gay/bisexual.

a *p* < .05.

b *p* < .01.

c *p* < .001.

### RQ2: Is age significantly associated with gender and sexual orientation in predicting orgasm rate?

The association between orgasm rate, gender, and sexual orientation was moderated by age. Age showed significant 2-way interactions with sexual orientation (code 2: gay/lesbian vs bisexual) and significant 3-way interactions with sexual orientation (code 2: gay/lesbian vs bisexual) and gender (women vs men). Simple effects tests indicated that age was positively associated with orgasm rate in bisexual men (*b* = 0.46, *t*_459_ = 5.25, *p* < .001, *r*_p_ = 0.24), lesbian women (*b* = 0.56, *t*_427_ = 4.97, *p* < .001, *r*_p_ = 0.23), and gay men (*b* = 0.30, *t*_1515_ = 5.38, *p* < .001, *r*_p_ = 0.14), with small effect sizes. There was no significant association for bisexual women (*b* = 0.20, *t*_883_ = 1.64, *p* = .10, *r*_p_ = 0.06).

The association between orgasm rate and sexual orientation (code 1: heterosexual vs gay/lesbian/bisexual) was moderated by the nonlinear age variable. Simple effects tests showed a nonlinear relationship between age and orgasm rates for heterosexual participants, with minimal effect size (*b* = −0.02, *t*_21435_ = −6.16, *p* < .001, *r*_p_ = −0.04). This effect was not significant for nonheterosexual participants (*b* = −0.01, *t*_3299_ = −0.85, *p* = .40, *r*_p_ = −0.02).

We then assessed sexual orientation and gender within 5 adult age groups. Neither sexual orientation nor its intersection with gender significantly predicted orgasm rates in emerging adults (18-24 years of age) or young adults (25-34 years of age). In early middle adults (35-49 years of age), late middle adults (50-64 years of age), and elder adults (65+ years of age), heterosexual participants reported higher orgasm rates than gay/lesbian/bisexual participants. This main effect was qualified by an interaction with gender, showing nuanced results. Heterosexual men had higher orgasm rates than nonheterosexual men in the late middle adult group (50-64 years of age), with a small effect size (*b* = −2.29, *t*_3144_ = −3.15, *p* < .01, *r*_p_ = −0.06). There were no significant differences for early middle adults (35-49 years of age) or elder adults (65+ years of age) (*b* = −1.33, *t*_3024_ = −1.66, *p* = .10, *r*_p_ = −0.03; and *b* = −0.94, *t*_1699_ = −0.84, *p* = .40, *r*_p_ = −0.02, respectively).

Among women, lesbian/bisexual women had higher orgasm rates in early middle adulthood (35-49 years of age) (*b* = 3.76, *t*_3393_ = 3.33, *p* < .001, *r*_p_ = 0.06). No significant differences were found in the late middle (50-64 years of age) or elder adult (65+ years of age) groups (*b* = 1.72, *t*_3402_ = 1.13, *p* = .26, *r*_p_ = 0.02 and *b* = 3.32, *t*_1697_ = 1.04, *p* = .30, *r*_p_ = 0.03, respectively).

Bisexual participants reported higher orgasm rates than gay/lesbian participants in late middle adulthood (50-64 years of age) (*b* = 9.12, *t*_6549_ = 2.16, *p* < .05, *r*_p_ = 0.03), with a small effect size. Significant 2-way interactions between sexual orientation (gay/lesbian vs bisexual) and gender were found in early middle adulthood (35-49 years of age) and late middle adulthood (50-64 years of age). In early middle adulthood (35-49 years of age), lesbian women reported higher orgasm rates than bisexual women (*b* = −7.50, *t*_3393_ = −3.46, *p* < .001, *r*_p_ = −0.06). No significant differences were found in late middle adulthood (50-64 years of age) for women (*b* = −4.15, *t*_3402_ = −1.39, *p* = .17, *r*_p_ = −0.02) or for men in either the early middle adulthood (35-49 years of age) or late middle adulthood (50-64 years of age) groups (*b* = −0.58, *t*3024 = −0.39, *p* = .70, *r*_p_ = −0.01; and *b* = 2.42, *t*_3144_ = 1.77, *p* = .08, *r*_p_ = 0.03, respectively).

### RQ3: Does the orgasm gap between men and women exist in all adult age groups?

In all 5 age groups, men reported higher rates of orgasm than women. Men’s orgasm rates ranged from 70% to 85%, while women’s rates ranged from 46% to 58%. Women’s reported orgasm rates were 22.43% (emerging adults) and 29.74% (elder adults) lower than men’s rates. See Table 3 for regression coefficients.

**Table 3:** Regression coefficients within each age group.

Variable	*b* (95% CI)	*t*	*r*_p_
Emerging adults (18-24 y)			
Income	1.04 (0.45 to 1.63)	3.44^c^	0.06
Year, linear	−1.53 (−2.05 to −1.00)	−5.67^c^	−0.10
Year, nonlinear	0.78 (0.51 to 1.04)	5.76^c^	0.10
Gender	−20.52 (−23.96 to −17.09)	−11.72^c^	−0.19
Sexual orientation: heterosexual vs LGB	−2.47 (−8.42 to 3.48)	−0.82	−0.01
Sexual orientation: gay/lesbian vs bisexual	3.66 (−7.40 to 14.73)	0.65	0.01
Gender × heterosexual vs LGB	2.85 (−0.58 to 6.29)	1.63	0.03
Gender × gay/lesbian vs bisexual	−3.09 (−9.42 to 3.24)	−0.96	−0.02
Young adults (25-34 y)			
Income	1.09 (0.59 to 1.59)	4.28^c^	0.06
Year, linear	−0.84 (−1.26 to −0.42)	−3.93^c^	−0.06
Year, nonlinear	0.57 (0.36 to 0.78)	5.38^c^	0.07
Gender	−25.29 (−28.18 to −22.39)	−17.13^c^	−0.24
Sexual orientation: heterosexual vs LGB	−2.04 (−6.57 to 2.48)	−0.89	−0.01
Sexual orientation: gay/lesbian vs bisexual	−3.91 (−12.31 to 4.50)	−0.91	−0.01
Gender × heterosexual vs LGB	2.05 (−0.84 to 4.94)	1.39	0.02
Gender × gay/lesbian vs bisexual	2.28 (−3.10 to 7.66)	0.83	0.01
Early middle adults (35-49 y)			
Income	0.80 (0.38 to 1.21)	3.78^c^	0.05
Year, linear	−0.59 (−0.94 to −0.24)	−3.28^c^	−0.04
Year, nonlinear	0.30 (0.13 to 0.47)	3.49^c^	0.04
Gender	−24.61 (−26.31 to −20.91)	−17.14^c^	−0.21
Sexual orientation: heterosexual vs LGB	−6.28 (−10.35 to −2.20)	−3.10^b^	−0.04
Sexual orientation: gay/lesbian vs bisexual	5.93 (−1.71 to 13.58)	1.52	0.02
Gender × heterosexual vs LGB	5.06 (2.37 to 7.75)	3.68^c^	0.05
Gender × gay/lesbian vs bisexual	−6.12 (−11.72 to −1.52)	−2.54^a^	−0.03
Late middle adults (50-64 y)			
Income	0.70 (0.32 to 1.08)	3.60^c^	0.04
Year, linear	−0.85 (−1.18 to −0.53)	−5.17^c^	−0.06
Year, nonlinear	0.17 (0.12 to 0.33)	2.10^a^	0.03
Gender	−25.33 (−28.49 to −22.18)	−15.76^c^	−0.19
Sexual orientation: heterosexual vs LGB	−6.32 (−10.65 to −1.98)	−2.86^b^	−0.04
Sexual orientation: gay/lesbian vs bisexual	9.12 (0.84 to 17.39)	2.16^a^	0.03
Gender × heterosexual vs LGB	3.98 (0.83 to 7.12)	2.48^a^	0.03
Gender × gay/lesbian vs bisexual	−6.63 (−12.71 to −0.55)	−2.14^a^	−0.03
Elder adults (65+ y)			
Income	0.85 (0.28 to 1.42)	2.91^b^	−0.50
Year, linear	−1.11 (−1.57 to −0.65)	−4.69^c^	−0.08
Year, nonlinear	0.24 (0.01 to 0.47)	2.03^a^	0.04
Gender	−22.44 (−27.93 to −16.95)	−8.02^c^	−0.14
Sexual orientation: heterosexual vs LGB	−10.97 (−17.45 to −4.48)	−3.31^c^	−0.06
Gender × heterosexual vs LGB	8.16 (2.68 to 13.64)	2.92^b^	0.05

For the elder adult group, no comparisons between bisexual and gay/lesbian participants were tested, due to lack of adequate statistical power. For emerging adults: *R*^2^ = 0.11, *R*^2^ adjusted = 0.11, *F*_8,3512_ = 52.21, *p* < .001. For young adults: *R*^2^ = 0.15, *R*^2^ adjusted = 0.15, *F*_8,4827_ = 104.42, *p* < .001.For early middle adults^:^*R*^2^ = 0.16, *R*^2^ adjusted = 0.16, *F*_8,6420_ = 157.79, *p* < .001. For late middle adults *R*^2^ = 0.19, *R*^2^ adjusted = 0.19, *F*_8,6549_ = 188.06, *p* < .001. For elder adults: *R*^2^ = 0.19, *R*^2^ adjusted = 0.19, *F*_6,3401_ = 135.33, *p* < .001.

Abbreviations: CI, confidence interval; LGB, lesbian/gay/bisexual.

a *p* < .05.

b *p* < .01.

c *p* < .001.

## Discussion

Using a cross-sectional sample of 24 752 U.S. adults, we explored the persistence of the orgasm gap across the adult lifespan, from emerging (18-24 years of age) to elder adulthood (65+ years of age). Overall, participants reported orgasming from sexual intercourse 65% of the time, with orgasm rates remaining relatively stable across age before accounting for differences by gender and sexual orientation. Results showed the orgasm gap persists across all age groups: men’s orgasm rates ranged from 70% to 85%, while women’s ranged from 46% to 58%. Men reported orgasm rates between 22% and 30% higher than women’s rates.

These findings align with previous research showing higher orgasm rates among men than women^12-14^ and highlight the stability of this gap with age. The presence of the orgasm gap across adulthood may be attributed to sociocultural influences and norms, such as the undervaluing of women’s sexual satisfaction, biased sexual education, and the emphasis on penetrative sex. These societal attitudes likely shape individuals’ sexual behaviors and expectations, perpetuating gendered dynamics favoring men’s pleasure across the lifespan. Further, inadequate inclusive sexual education overlooks mutual pleasure^61^ and may reinforce attitudes and behaviors that uphold the orgasm gap.

Our findings on age-related orgasm rates, particularly among different sexual orientations and genders, highlight the complexity of sexual satisfaction. Older gay and bisexual men and lesbian women reported higher orgasm rates than their younger counterparts, suggesting that age may enhance understanding of one’s own sexual needs and preferences. However, the persistent gap between men and women across all ages indicates that age alone does not address the underlying factors contributing to orgasm disparities.

This study also highlights the critical need to include sexual orientation in discussions about sexual pleasure and aging. Sexual minorities are often underrepresented in gerontological research. Studies on lesbian aging indicate that, in response to societal marginalization, these individuals may develop resilient strategies to enhance their well-being, potentially prioritizing sexuality.^62^^,^^63^ In late middle adulthood (50+ years of age), gay and bisexual men reported lower orgasm rates compared with heterosexual men, contrasting with similar rates in younger cohorts. Emerging research suggests that gay men may face unique aging-related stigma, which, combined with physiological aging, could impact sexual opportunities and experiences.^64^ Further research is needed to explore sexual pleasure and well-being among aging sexual minorities to address these disparities.

### Clinical implications

The findings on the association between age and the orgasm gap have significant clinical implications for healthcare providers, therapists, and sex educators. The persistence of the orgasm gap, influenced by sociocultural norms and insufficient sexual education, calls for a more inclusive approach to sexual health discussions and interventions. Clinicians should recognize the diverse experiences of sexual pleasure among different genders and sexual orientations and the unique challenges faced by sexual minorities, especially as they age. Tailoring sexual health education to address the full spectrum of sexual experiences and emphasizing mutual satisfaction and communication could help mitigate the orgasm gap. Additionally, recognizing and addressing the specific needs and challenges of older adults, particularly sexual minorities, is crucial. This involves creating safe spaces for discussions about aging and sexuality, combating stigma, and providing resources to support a fulfilling sexual life into later adulthood. A nuanced understanding of the factors contributing to the orgasm gap can inform targeted interventions to promote sexual well-being for all individuals, regardless of age, gender, or sexual orientation.

### Limitations, future directions, and conclusion

Results of this study have notable limitations. The survey had relatively small participation from sexual minorities, particularly in older age groups, limiting some comparisons. The smaller proportion of sexual minority participants also precluded further intersectional analyses with respect to race or ethnicity. Future studies should oversample sexual minority adults, especially in middle and elder adulthood, and aim for larger samples of people of color, as racial differences in the orgasm gap have been observed.^16^

Further, it is important to acknowledge the limitations of the orgasm rate measurement used in this research. Orgasm rates reported here are based on an unvalidated, single-item measure. While single-item measures can demonstrate considerable validity and reliability compared with multi-item measures,^65^^,^^66^ they lack the ability to capture the nuance of participants’ experiences in a multifaceted fashion. Additionally, the absence of validation and reliability testing for this specific measure raises concerns about its appropriateness and suitability for accurately assessing orgasm rates. Future research should incorporate validated and reliable measures of orgasm rate to improve the accuracy and generalizability of findings. Moreover, the cross-sectional nature of the survey prevents tracking individual changes over time. Future researchers should design longitudinal panel studies to observe how specific individuals’ orgasm rates change over their lifespan.

In conclusion, this study confirms the persistent orgasm gap throughout the adult lifespan, tied to a complex web of physiological, psychological, and sociocultural factors. Our findings align with prior literature, showing variability in orgasm rates by gender and sexual orientation. Despite the richness of our data, methodological limitations such as inadequate diversity in participants’ sexual orientation and relationship status, and reliance on cross-sectional and single-item assessments, highlight the need for future longitudinal studies with intentional recruitment around participants’ identities. Such efforts would provide a more detailed understanding of orgasm rates across the lifespan and pave the way for interventions aimed at bridging the orgasm gap.

## Data Availability

Supplemental materials including dataset and syntax are available at https://osf.io/xyg6e/.
